# The necessity of integrated medicine to treat SARS‐Cov‐2/COVID‐19 patient: A case report

**DOI:** 10.1002/ccr3.5041

**Published:** 2021-11-06

**Authors:** Sagun Tiwari, Sujan Tiwari, Namrata Sapkota, Bhanu Sapkota

**Affiliations:** ^1^ Department of Neurology and Rehabilitation Seventh People's Hospital of Shanghai University of TCM Shanghai China; ^2^ Shanghai University of TCM Shanghai China; ^3^ Tribhuvan University Kirtipur Nepal; ^4^ Net Fresh Hospital Bharatpur Metropolitan City Nepal; ^5^ Om Wellness Hospital Biratnagar Nepal; ^6^ Armed Forces Medical College Dhaka Bangladesh

**Keywords:** COVID‐19, home treatment, integrated medicine, naturopathy, SARS‐CoV‐2, Traditional Chinese Medicine, yoga

## Abstract

Health policymakers and clinicians should also prioritize mental, emotional and social health while treating SARS‐CoV‐2/COVID‐19, and for this, different treatment varieties of eastern medicine would be a better option to integrate into western medicine so that we could have a better result in all dimensions of health.

## Graphical abstract

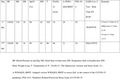

## 1 BACKGROUND

Psychological and psychosocial issues are more prominent issues of an individual in any disease, which could further deteriorate patients' condition and hamper their quality of life. However, in treating SARS‐CoV‐2/COVID‐19,those patients'mental, social, and emotional aspects are still overlooked.

SARS‐Cov‐2 is currently one of the deadliest diseases globally, with high rates of infection, incidence, and fatality, and the whole public is at risk.^1^, ^2^ Since its beginning, most governments have used severe constraints to break the chain of transmission, such as lockdowns, curfews, and so on. However, managing COVID‐19/SARS‐Cov‐2 cases worldwide remains challenging due to a lack of vaccine availability, healthcare staff, and healthcare supplies, especially in low‐income countries. In addition, in a developing country like Nepal, where vaccine production is non‐existent (current vaccination rate is still below 20%), the doctor‐to‐patient ratio is 0.17 per 1,000 people, and medical facilities and supplies are scarce, the country is facing medical emergency‐like conditions throughout this pandemic.^3^ Fever, cough, headache, dyspnea, chest pain, and other symptoms are common in SARS‐CoV‐2 patients.

Aside from its physical health consequences, the epidemic has been linked to considerable social and emotional stress because of the extent to which it has impacted daily living and the general public's quality of life.^4^ Until recently, everyone has been concerned with treating COVID patient's physical signs and symptoms only, but no one has been concerned about an individual's overall health, that is, physical, mental, emotional, and social. According to the World Health Organization, total health encompasses physical, mental, and social well‐being, not just the absence of disease or disability. However, in a nation like Nepal, few people are aware of their mental and social health and how it affects their daily lives, and the only thing that really matters to most is to get rid of physical symptoms. SARS‐CoV‐2 also exhibits various stress‐related health problems, such as sleeplessness, depression, anxiety and panic attacks, cerebrovascular illness, and so on. However, in the case of SARS‐CoV‐2, great emphasis has been placed on an individual's physical well‐being, while other aspects of health have received scant medical attention. Study demonstrates that eastern medicine helps promote an individual's mental and social well‐being by lowering stress, anxiety, and even helping to improve physical well‐being, quality of life, and immunity.^5^, ^6^, ^7^, ^8^, ^9^, ^10^ That is why, in order to manage all areas of health in SARS‐CoV‐2 positive patients, we blended eastern and western medicine (physical, mental, and social). To our knowledge, there has not been any research investigating using both eastern and western medicine to treat SARS‐Cov‐2 patients in Nepal in order to manage all aspects of their health. This case study intends to describe the potential benefits of combining eastern and western medicine in SARS‐Cov‐2 patients in order to assist health policymakers in implementing an integrated medicinal approach to treat SARS‐Cov‐2 patients effectively.

## 2 CASE PRESENTATION

A 49‐year‐old woman contacted a health professional via virtual video call with symptoms of fever, cough, running nose, sore throat, and tiredness for 1 day. Following a virtual interview, onsite medical personnel was referred to the participant's house for a face‐to‐face interview and acquisition of initial parameters, which were as follows: blood pressure (BP),130/82 mm/Hg; heart rate (HR), 110 beats/min; respiratory rate (RR), 18 breaths/min; SpO2 level, 98%; body weight(W), 62 kg, and body temperature (T), 103 ^O^F; DASS‐21 Score,34; A‐WHOQOL‐BREF score,28; PSS‐10‐C score,29. The patient was dispatched to give a nasopharyngeal swab on the same day to confirm the SARS‐Cov‐2 diagnosis, and on the next day, results came, confirming the diagnosis of SARS‐CoV‐2/COVID‐19.

With her signed consent, a combined treatment of western and eastern medicine was used as an interventional approach to the participants (Table 1). Treatment and/or advice was provided either in person or via virtual video call. We kept track of the patient's vitals and observed her health on a daily basis. In addition, on the first and last days of the SARS‐Cov‐2 test, health‐related questionnaires were collected (ie, 17th day). Day by day, her condition got better as a result of the intervention we put in place for her. Finally, on the 17th day, the patient had a COVID negative report without any accompanying symptoms, and her DASS‐21, A‐WHO‐QoL, and PSS‐10‐C scores were all altered by 30, 3, and 5, respectively (Table 2), implying better quality of life and overall health.

**TABLE 1:** Integrated (Western and Eastern Medicine) treatment and/or guidance for COVID/SARS‐Cov‐2 patients

Day	Morning	Afternoon	Evening
1	‐	‐	Yoga (Pranayama, Meditation), Hot foot immersion with a cold compress on the head, Metronidazole−400 mg, Fexofenadine‐ 180 mg, Azithromycin−500 mg
2	Yoga (Asana, Pranayama), Jala Neti followed by Kapalbhati (10 strokes), Steam inhalation with *Ocimum tenuiflorum*, peppermint and eucalyptus oil, Metronidazole−400 mg	Acupressure, Counseling, Metronidazole−400 mg	Yoga (Pranayama, Meditation), Hot foot immersion with a cold compress on the head, Steam inhalation with *Ocimum tenuiflorum*, peppermint and eucalyptus oil, Metronidazole−400 mg, Fexofenadine‐ 180 mg, Azithromycin−500 mg
3	Yoga (Asana, Pranayama), Jala Neti followed by Kapalbhati (10 strokes), Steam inhalation with *Ocimum tenuiflorum*, peppermint and eucalyptus oil, Metronidazole−400 mg	Acupressure, Counseling, Metronidazole−400 mg	Yoga (Pranayama, Meditation), Hot foot immersion with a cold compress on the head, Steam inhalation with *Ocimum tenuiflorum*, peppermint and eucalyptus oil, Metronidazole−400 mg, Fexofenadine−180 mg, Azithromycin−500 mg
4	Yoga (Asana, Pranayama), Jala Neti followed by Kapalbhati (10 stroke), Metronidazole−400 mg	Acupressure, Counseling, Metronidazole−400 mg	Yoga (Pranayama, Meditation), Hot foot immersion with a cold compress on the head; Steam inhalation with *Ocimum tenuiflorum*, peppermint and eucalyptus oil, Metronidazole−400 mg, Fexofenadine‐ 180 mg, Azithromycin−500 mg
5	Taichi, Jala Neti followed by Kapalbhati (10 strokes), Steam inhalation with *Ocimum tenuiflorum*, peppermint and eucalyptus oil, Metronidazole−400 mg	Acupressure, Counseling, Metronidazole−400 mg	Yoga (Pranayama, Meditation), Hot foot immersion, Metronidazole−400 mg, Fexofenadine‐ 180 mg, Azithromycin−500 mg
6	Yoga (Asana, Pranayama), Jala Neti followed by Kapalbhati (10 stroke), Metronidazole−400 mg	Acupressure, Counseling, Metronidazole−400 mg	Yoga (Pranayama, Meditation), Hot foot immersion, Metronidazole−400 mg, Fexofenadine‐ 180 mg, Azithromycin−500 mg
7	Yoga (Asana, Pranayama), Jala Neti followed by Kapalbhati (10 stroke)	Acupressure, Counseling	Yoga (Pranayama, Meditation), Hot foot immersion
8	Taichi, Jala Neti followed by Kapalbhati (10 strokes)	Acupressure, Counseling	Yoga (Pranayama, Meditation), Hot foot immersion
9	Yoga (Asana, Pranayama), Steam inhalation with *Ocimum tenuiflorum*, peppermint and eucalyptus oil	Acupressure, Counseling	Yoga (Pranayama, Meditation), Hot foot immersion
10	Taichi, Jala Neti followed by Kapalbhati (10 strokes)	Acupressure, Counseling	Yoga (Pranayama, Meditation), Hot foot immersion
11	Yoga (Asana, Pranayama), Steam inhalation with *Ocimum tenuiflorum*, peppermint and eucalyptus oil	Acupressure, Counseling	Yoga (Pranayama, Meditation), Hot foot immersion
12	Taichi, Jala Neti followed by Kapalbhati (10 strokes)	Acupressure, Counseling	Yoga (Pranayama, Meditation), Hot foot immersion
13	Yoga (Asana, Pranayama), Steam inhalation with *Ocimum tenuiflorum*, peppermint and eucalyptus oil	Acupressure, Counseling	Yoga (Pranayama, Meditation), Hot foot immersion
14	Taichi, Steam inhalation with *Ocimum tenuiflorum*, peppermint and eucalyptus oil	Acupressure, Counseling	Yoga (Pranayama, Meditation)
15	Yoga (Asana, Pranayama), Jala Neti followed by Kapalbhati (10 stroke)	Acupressure, Counseling	Yoga (Pranayama, Meditation)
16	Taichi, Steam inhalation with *Ocimum tenuiflorum*, peppermint and eucalyptus oil	Acupressure, Counseling	Yoga (Pranayama, Meditation)
17	Yoga (Asana, Pranayama)	Acupressure	Taichi

**TABLE 2:** Progress report of the patient

Day	BP	HR	RR	SpO2 (%)	BW	T	DASS−21 Score	A‐WHO‐QOLBREF	PSS−10‐C	SARS‐Cov−2 Test—Real‐Time RT PCR	Remarks
1^st^ day	130/82	110	18	98	62	103	34	28	29	Positive	E‐Gene Ct Value:21.32 RdRp‐Gene Ct Value: 22.58 N‐Gene Ct Value:22.33
17^th^ day	120/88	80	16	99	65	98	06	31	24	Negative	

Abbreviations: A‐WHOQOL‐BREF, Adapted version WHOQOL‐BREF to assess QoL in the context of the COVID‐19 pandemic; BP, Blood Pressure in mm/Hg; BW, Body Weight in kg; DASS‐21, The Depression, Anxiety and Stress Scale‐ 21; HR, Heart Rate in beats/min; PSS‐10‐C, Pandemic‐Related Perceived Stress Scale of COVID‐19; RR, Respiratory Rate in breaths/min; T, Temperature in ^o^F.

## 3 DISCUSSION

Numerous studies have shown that an integrated medicine strategy can improve outcomes not just for physical illnesses but also for mental and social dimensions of an individual in many diseases.^11^, ^12^, ^13^ Many scholars believe that integrated medicine is the future of medicine since it can address all dimensions of human health problems and enhance people's quality of life and disease prognosis.^14^, ^15^, ^16^ However, little emphasis has been paid to evaluating and assessing both eastern (traditional Chinese medicine, yoga, naturopathy, and Ayurveda) and western therapies, that is, integrated medicine, in managing SARS‐CoV‐2 patients in all dimensions of health, including physical, mental, and social. Based on our knowledge, our study is the first study to evaluate the effect of an integrated medicinal approach on SARS‐CoV‐2/COVID‐19 patients in Nepal. Every day, we checked the patient's vital signs, such as temperature, SpO2, RR, and so on. In addition, on the 1st and 17th days, the participants' mental status and quality of life were assessed using the DASS‐21, A‐WHO‐QoL, and PSS‐10‐C questionnaires.

People throughout the world are experiencing higher psychological stresses/challenges as a result of the COVID‐19 epidemic. Fear of contracting the coronavirus, the death of loved ones, economic and occupational crisis, and other psychosocial issues, as well as a sense of impending doom, are all weighing heavily on people's minds. Mental health professionals must precisely measure the burden of psychological and psychosocial difficulties in the community in order to provide quick psychological first‐aid to persons in need. Structured and proven tools will indeed be necessary to address the themes as mentioned earlier adequately. DASS‐21, A‐WHO‐QoL, and PSS‐10‐C questionnaires are a few of the validated and well‐proven questionnaires that we employed to evaluate the patients' mental status and quality of life in our study.^17^, ^18^, ^19^

Our findings suggest that treating SARS‐CoV‐2 patients with asymptomatic to moderate symptoms with an integrated medicinal approach in a quarantined setting can result in better outcomes and a better prognosis, which could benefit developing countries with low doctor‐to‐patient ratios and limited medical infrastructure/equipment. Furthermore, patients' anxiety and tension may be better managed with this technique, as eastern medicine provides an additional force that aids the conventional therapy approach. As a result, there would be less of a chance that the patient would not receive conventional care or therapy. Furthermore, the treatments/methods/guidance used in eastern medicine have nearly no negative effects. As a result, our research could serve as a wake‐up call for health policymakers to develop an integrated medical system to manage SARS‐CoV‐2 patients successfully.

In particular, to our knowledge, this is the first study in Nepal to look into the efficiency of both eastern and western medical interventions in a patient with SARs‐Cov‐2, one of the deadliest diseases that has harmed a person's physical, emotional, and social well‐being. At the end of the 17^th^ day, the patient felt that this type of integrated medicine had greatly enhanced her emotional and physical well‐being altogether and suggested implementing an integrated treatment strategy to other SARS‐Cov‐2/COVID‐19 patients. Our findings show that combining eastern and western treatment can improve patients' overall quality of life by eradicating all symptoms, lowering tension and anxiety, and enhancing their overall quality of life. However, there are some drawbacks to be aware of our study. First, we cannot speak for the large‐scale population to handle SARS‐Cov‐2 with this strategy because it is merely a case study. Large‐scale studies should be conducted in the future to determine whether integrated therapy modalities are beneficial to patients in the long run and whether they continue to be utilized to improve patients' overall quality of life. Second, there are no particular guidelines to incorporate both systems into one and utilize into patients with SARs‐Cov‐2/COVID‐19 patients. Thus, it is better to have a systemic treatment protocol to handle patients with SARs‐Cov‐2/COVID‐19 by integrated medicine.

## CONFLICTS OF INTEREST

The authors have no conflicts of interest to declare.

## AUTHOR CONTRIBUTIONS

All authors: Conceptualization and design, Administrative support, Provision of study materials, Collection, assembly, and interpretation of data, Manuscript writing, and Final approval of manuscript.

## ETHICAL APPROVAL

The case report was approved by the institute's ethical committee, and written informed consent was obtained from the patients for the participation of this study.

## CONSENT

Written informed consent was obtained from the patients for the publication of this study.

## Data Availability

*Data*/*Reports* are *available* from the authors upon reasonable *request* and with the patient's permission.
